# The natural breakthrough: phytochemicals as potent therapeutic agents against spinocerebellar ataxia type 3

**DOI:** 10.1038/s41598-024-51954-3

**Published:** 2024-01-17

**Authors:** Muhammad Naveed, Nouman Ali, Tariq Aziz, Nimra Hanif, Mahnoor Fatima, Imran Ali, Metab Alharbi, Abdullah F. Alasmari, Thamer H. Albekairi

**Affiliations:** 1 Department of Biotechnology, Faculty of Science and Technology, University of Central Punjab, Lahore, 54590 Pakistan; 2 Laboratory of Animal Health, Food Hygiene and Quality, Department of Agriculture, University of Ioannina, 47100 Arta, Greece; 3 Department of Pharmacology and Toxicology, College of Pharmacy, King Saud University, P.O. Box 2455, 11451 Riyadh, Saudi Arabia

**Keywords:** Biochemistry, Computational biology and bioinformatics, Drug discovery

## Abstract

There is no FDA-approved drug for neurological disorders like spinocerebellar ataxia type 3. CAG repeats mutation in the ATXN3 gene, causing spinocerebellar ataxia type 3 disease. Symptoms include sleep cycle disturbance, neurophysiological abnormalities, autonomic dysfunctions, and depression. This research focuses on drug discovery against ATXN3 using phytochemicals of different plants. Three phytochemical compounds (flavonoids, diterpenoids, and alkaloids) were used as potential drug candidates and screened against the ATXN3 protein. The 3D structure of ATXN3 protein and phytochemicals were retrieved and validation of the protein was 98.1% Rama favored. The protein binding sites were identified for the interaction by CASTp. ADMET was utilized for the pre-clinical analysis, including solubility, permeability, drug likeliness and toxicity, and chamanetin passed all the ADMET properties to become a lead drug candidate. Boiled egg analysis attested that the ligand could cross the gastrointestinal tract. Pharmacophore analysis showed that chamanetin has many hydrogen acceptors and donors which can form interaction bonds with the receptor proteins. Chamanetin passed all the screening analyses, having good absorption, no violation of Lipinski’s rule, nontoxic properties, and good pharmacophore properties. Chamanetin was one of the lead compounds with a − 7.2 kcal/mol binding affinity after screening the phytochemicals. The stimulation of ATXN3 showed stability after 20 ns of interaction in an overall 50 ns MD simulation. Chamanetin (Flavonoid) was predicted to be highly active against ATXN3 with good drug-like properties. *In-silico* active drug against ATXN3 from a plant source and good pharmacokinetics parameters would be excellent drug therapy for SC3, such as flavonoids (Chamanetin).

## Introduction

Machado-Joseph disease (MJD), or type 3 spinocerebellar ataxia, is most common in people of Japanese, European, and American ancestry^1^. Recent systematic reviews estimate that 100 out of every 100,000 people have SCA. The neurodegenerative and dominantly inherited disease most prevalent in the world is spinocerebellar ataxia type 3, or SCA3. Expansion of CAG repeats encoding polyglutamine (polyQ) in the ATXN3 gene encoding the deubiquitinating enzyme^2^. In both infected and normal individuals, the chromosomal location of this gene is the same, and they only differ in repeats. SCA arises when progressive degeneration occurs in the cerebellum and affects all interconnected brain regions^3^. All the Diseases related to polyQ are age-relevant developing disorders that start in midlife and cause death after 15–30 years of occurrence^2^. SCA3 clinical symptoms are the passive stance, limb ataxia, gait, dystonia, dysarthria, intentional tremor, bradykinesia, oculomotor disorders, pyramidal, sensory deficits, extrapyramidal dysfunctions, sleep disturbances, neurophysiological abnormalities, autonomic dysfunctions and depression^1^. In neurons of the SCA3 and neurodegenerative diseases, ubiquitin-positive nuclear inclusions occur due to the common neuropathological hallmark. The polyQ is disease-related and plays an essential role in transcriptions, leading to dying expansions by causing the disturbance in transcriptional profiles and normal functions of the nervous system.

The structure and function of the ATXN3 protein is pivotal. ATXN3 is a 42-kDa protein with high brain and spinal cord expression levels. It exists in two primary splice variants, exhibiting variations at the carboxyl terminus^4^. While Ataxin-3 predominantly localizes within the nuclei of neurons in Machado-Joseph Disease (MJD), this small soluble protein can shuttle between the nucleus and cytoplasm. As a deubiquitinating enzyme, Ataxin-3 is responsible for cleaving polyubiquitin chains. Its distinctive polyglutamine tract, exclusive to humans, and the Josephin domain further support its role. The Josephin domain contains a predicted catalytic amino acid triad akin to ubiquitin-specific cysteine proteases^5,6^. To understand the pathophysiology and progression of Spinocerebellar Ataxia Type 3 (SCA3), it is crucial to grasp the intricate structure and multifaceted functions of the ATXN3 protein. This clarification serves as the groundwork for future research endeavors into the molecular processes of the disease, providing a basis for potential therapeutic interventions aimed at alleviating its devastating effects.

Recent advancements in next-generation sequencing helped find and identify the new genes involved in the pathogenesis and transmission of the disease^3^. In comparative studies, central nervous system grey matter degeneration affection is more severe than the white matter in the case of the SCA3. In the case of SCA3, specific white matter structures, such as the cerebellar white matter, cranial nerves, cerebellar peduncles, trapezoid body, medial lemniscus, vestibulospinal, medial longitudinal, spinocerebellar, spinothalamic tracts, gracile fascicles, and cuneate face, exhibit myelin loss. In SCA3 disorder, neurotransmitters and many functional systems are highly affected by the pathological processes of the SCA3 disease. Genetical counseling is fundamental in pre-implantation and the prevention of diseases. Gene silencing is an effective method to implant disease markers before time^7^. Artificial microRNA is engineered to target the TXN3. AAV5-miATXN3 treatment is performed against the targeted site. Patients with SCA3 can benefit from the transduction activity of AAV5. These findings established a solid foundation for further research into the distribution, efficacy, and safety of AAV5-miATXN3 in large animals despite having some undesirable side effects, such as dizziness and loss of appetite^8,9^.

This study aims to design a phytochemical-based drug using computational approaches to treat spinocerebellar ataxia type 3 disease. Designing in silico drugs is to create and discover novel medications or improve existing ones using computer-based methods and simulations. In general, in silico drug design speeds up the process of finding new drugs, minimizes the number of substances that must be created and tested in experiments to check their toxic and allergenic effects and offers important new information on the molecular causes of disease and drug interactions.

## Materials and methods

### Retrieval of targeted protein

The tertiary structure of ATXN3 protein (PDB ID: 1YZB) was retrieved from the Protein Data Bank (https://www.rcsb.org/)^10^. RCSB PDB data had 3D structures of proteins experimentally constructed by X-ray crystallography technique.

### 3D structure validation

The tertiary structure of the ATXN3 protein was validated using Procheck Ramachandran Plot^11^. Ramachandran’s plot was constructed to check the torsion angle distribution of the protein residues on psi and phi on a 2D plane.

### Binding site identification

The active sites were identified using CastP (http://sts.bioe.uic.edu/castp/index.html?2pk9)^12,13^. The binding site is also known as the protein's pocket or active site, and it’s the site where the small molecule (ligand) interacts with the protein.

### Identification of ligands

Three compounds, flavonoids, diterpenes, and alkaloids, were selected from the different plant sources available in the literature as ligands for screening purposes. The 3D models were downloaded in SDF format from PubChem (https://pubchem.ncbi.nlm.nih.gov/)^14^.

### Screening of compounds

Compounds from flavonoids, diterpenes and alkaloids were screened through Pyrx^15^. PyRx was a virtual screening software for computational drug discovery and development for searching the inhibitors for diseases in the emerging world. For screening, the receptor was converted into a pdbqt file and then all ligands’ energies were minimized to convert them into a pdbqt file. The grid box was set on the amino acids of the pocket detected by the CASTp. Among all these, the compound with the highest energy was selected for further optimizations to increase its efficiency.

### Pharmacophore characterization

Pharmacophore characterization of the ligand was performed by the pharmit server (https://pharmit.csb.pitt.edu/search.html)^16,17^. The bonds between the ligand and its ability to accept and donate hydrogens were checked.

### ADMET analysis

The SwissADME tool was used for the pre-clinical testing of a drug candidate (http://www.swissadme.ch/)^18^. This computational tool predicted pharmacokinetic properties. The medicinal properties and drug nature properties of drug candidates are also measured. Drug likeliness was calculated from the molinspiration cheminformatics server (https://www.molinspiration.com)^19^. The toxicity of the ligand was analyzed by the Pro Tox II server (https://tox-new.charite.de/protox_II/index.php?site=home)^17,20^.

### Molecular interaction analysis

Autodock Vina, the open-source software for docking analysis of the selected best-screen active compounds, was used^21,22^. The pdbqt files for the protein and ligands were created utilizing MGL tools. In the protein pdbqt, polar hydrogens were incorporated. The adjustment of the protein's binding site involved utilizing a grid box on the active residues, determined by CASTp with x (− 7.802), y (− 0.818), and z (− 1.580) values having size 16. The ligand's torsion angle was set at 6. Subsequently, 9 conformations of the ligand with the receptor were generated, and the most optimal conformation was selected for in-depth molecular interaction studies. To analyze the molecular interaction, Bio-discovery Studio, PyMol and ligplot + were further used to visualize the Protein–ligand interacted complex and 3D and 2D interaction diagrams were constructed^23,24^.

### MD simulations

GROMACS was used for the MD simulation, run from the UMAS web grow server (https://simlab.uams.edu/)^25,26^. GROMOS96 54a7 Forefield, water model SPC, Triclinic box and NaCl were used, and a 50 ns simulation was performed on the best dock complex. RSMD and RSMF were calculated for ligand and protein.

## Results

### Retrieval of the targeted protein

**Figure 1:**
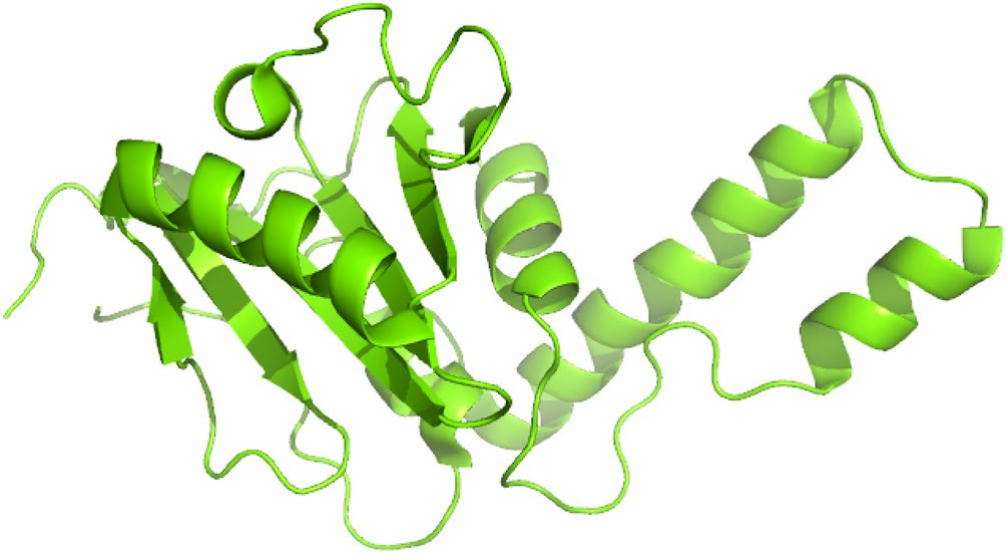
3D structure of ATXN3 Protein.

ATXN3 receptor protein 3D structure was retrieved from PDB under PDB ID 1YZB. The structure was obtained through Nuclear Magnetic Resonance (NMR) technology, and the expression system employed was Escherichia coli BL21 (DE3) with a single chain. The 3D structure of the ATXN3 protein can be seen in Fig. 1.

### Validation of the tertiary structure of the protein

**Figure 2:**
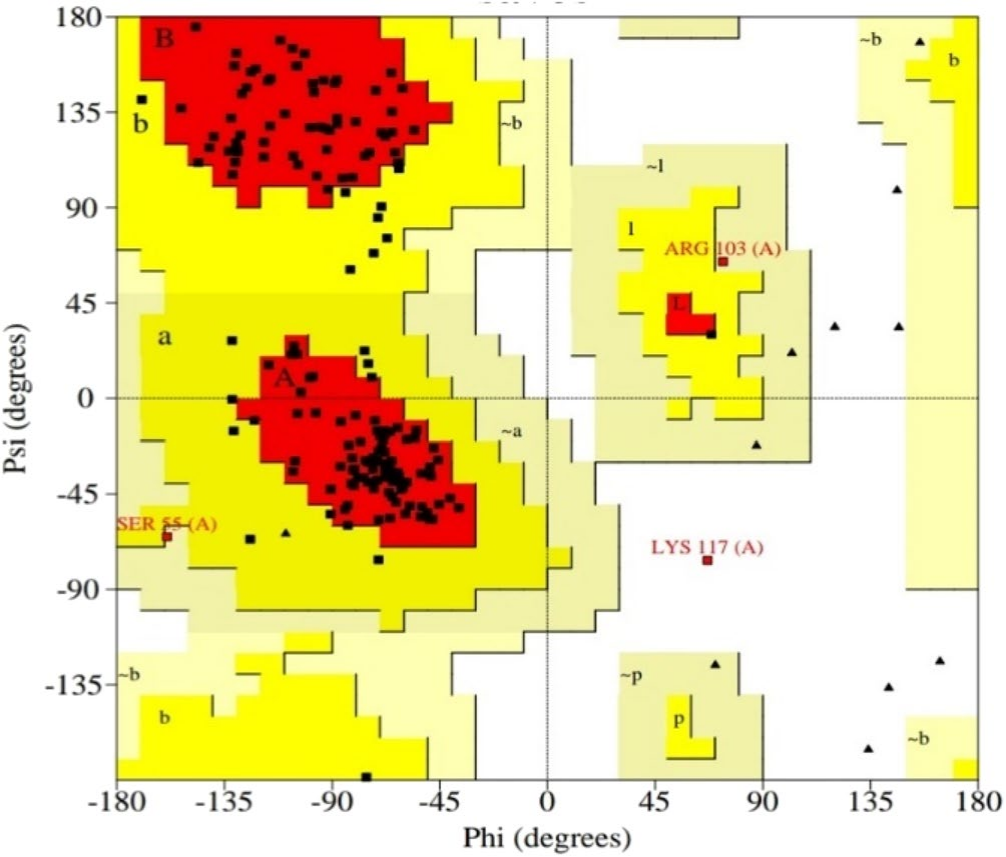
Ramachandran plot of ATXN3 3D structure shows the residues in Ramachandran favored regions. The red-colored region is the most favorable, and yellow is the favorable region.

The Ramachandran plot showed an excellent score, with the most favored Rama score (favorable and most favorable region both) of 98.1 within 160 amino acid residues. Ramachandran's plot can be seen in Fig. 2**.**

### Binding site identification

**Figure 3:**
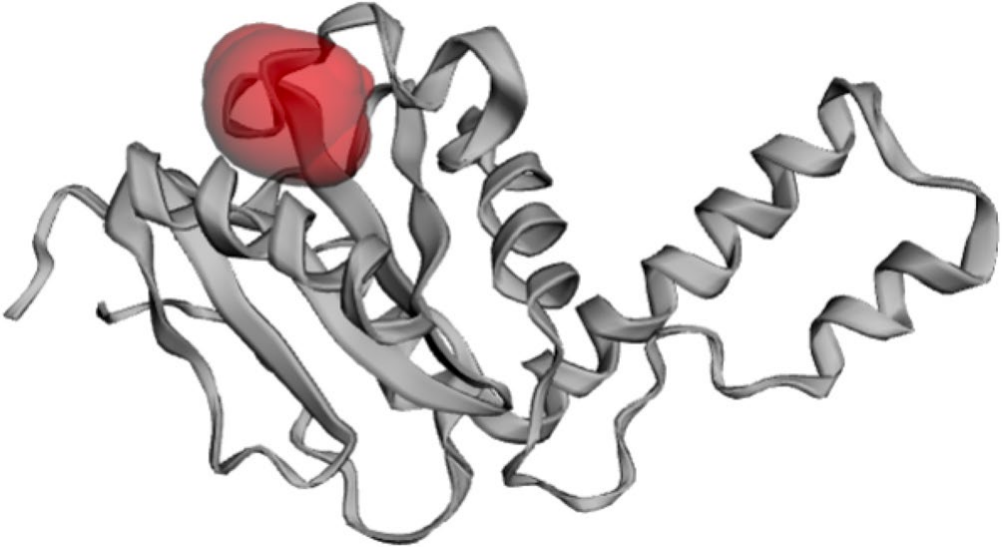
Active site prediction by CASTp. Red highlights are active site pockets.

CASTP was used for the analysis of binding sides. The three-dimensional regions were marked, delineated, and measured. As in the protein 1yzb, Richards' surface (molecular surface or solvent-excluded surface) and Connolly's surface (solvent-accessible surface area) are fully visible and marked red; at this point, the ligands can fully interact with the protein pockets. The active site of the ATXN3 protein can be seen in Fig. 3**.** The residues in the pocket were Phe94, Tyr99, Gln100, Arg101, Leu102, Arg103, Ile104, Pro106, Ala149, Leu152, Ala153 and Gln156.

### Identification of ligands

For screening purposes, flavonoids, alkaloids and diterpenes were chosen from the literature in Table 1. The 3D structures were retrieved from PubChem in SDF format and then saved in PDB format using Discovery Studio for further analysis.

**Table 1:** Phytochemical PubChem CIDs.

Sr. No.	Phytochemicals	PubChem CID
Flavonoids		
1	Chamanetin	21721821
2	Cyclocommunol	10315987
3	Lupinifolin	10250777
4	Neobavaisoflavone	5320053
5	8-Diprenyleriodictyol	4063836
6	6-Prenylpinocembrin	480763
7	Brazilin	73384
8	Quercetin	280343
9	Myricetin	5281672
10	Candidone	157102
Diterpenoids		
11	Teucrin	159529
12	Dehydroabietic acid	94391
13	Carnosol	442009
14	Bafoudiosbulbin	101410701
15	Lagascatriol	10448831
16	Aethiopinone	157301
17	Ferruginol	442027
18	Carnosic acid	65126
19	19-Dihydroxyserrulat-14-ene	23642601
Quinolines		
20	Ofloxacin	4583
21	Laquinimod	54677946
22	Pamaquine	10290
23	Evocarpine	5317303
24	4-Methylquinoline	10285
25	8-Hydroxyquinoline	1923
26	Clioquinol	788

### Screening of ligands

All the flavonoids, diterpenes and alkaloids were docked against the 1yzb protein using PyRx To filter out docking energies of the best drugs. The docking energies of flavonoids range from − 5.9 to − 7.2 kJ/mol; diterpenoids binding energies range from − 7 to − 5.4 kJ/mol, and quinolines range from − 6.9 to − 5.1 kJ/mol given in Table 2. Chamanetin from flavonoids was the best candidate since it had the lowest binding affinity with the protein, roughly − 7.2. The binding energies of all ligands can be seen in Table 2.

**Table 2:** Screen of ligands through autodock vina using pyrx.

Sr. No.	Ligands	Binding Energies KJ/mol
Flavonoids		
1	Chamanetin	− 7.2
2	Cyclocommunol	− 6.9
3	Lupinifolin	− 6.9
4	Neobavaisoflavone	− 6.7
5	8-Diprenyleriodictyol	− 6.8
6	6-Prenylpinocembrin	− 6.4
7	Brazilin	− 6.2
8	Quercetin	− 6.2
9	Myricetin	− 6.1
10	Candidone	− 5.9
Diterpenoids		
11	Teucrin	− 7.0
12	Dehydroabietic_acid	− 6.6
13	Carnosol	− 6.5
14	Bafoudiosbulbin	− 6.3
15	Lagascatriol	− 6.3
16	Aethiopinone	− 6.1
17	Ferruginol	− 6.0
18	Carnosic_acid	− 6.0
19	19-Dihydroxyserrulat-14-ene	− 5.4
Quinolines		
20	Ofloxacin	− 6.9
21	Laquinimod	− 6.4
22	Pamaquine	− 5.6
23	Evocarpine	− 5.4
24	4-Methylquinoline	− 5.3
25	8-Hydroxyquinoline	− 5.2
26	Clioquinol	− 5.1

### Pharmacophore characterization

**Figure 4:**
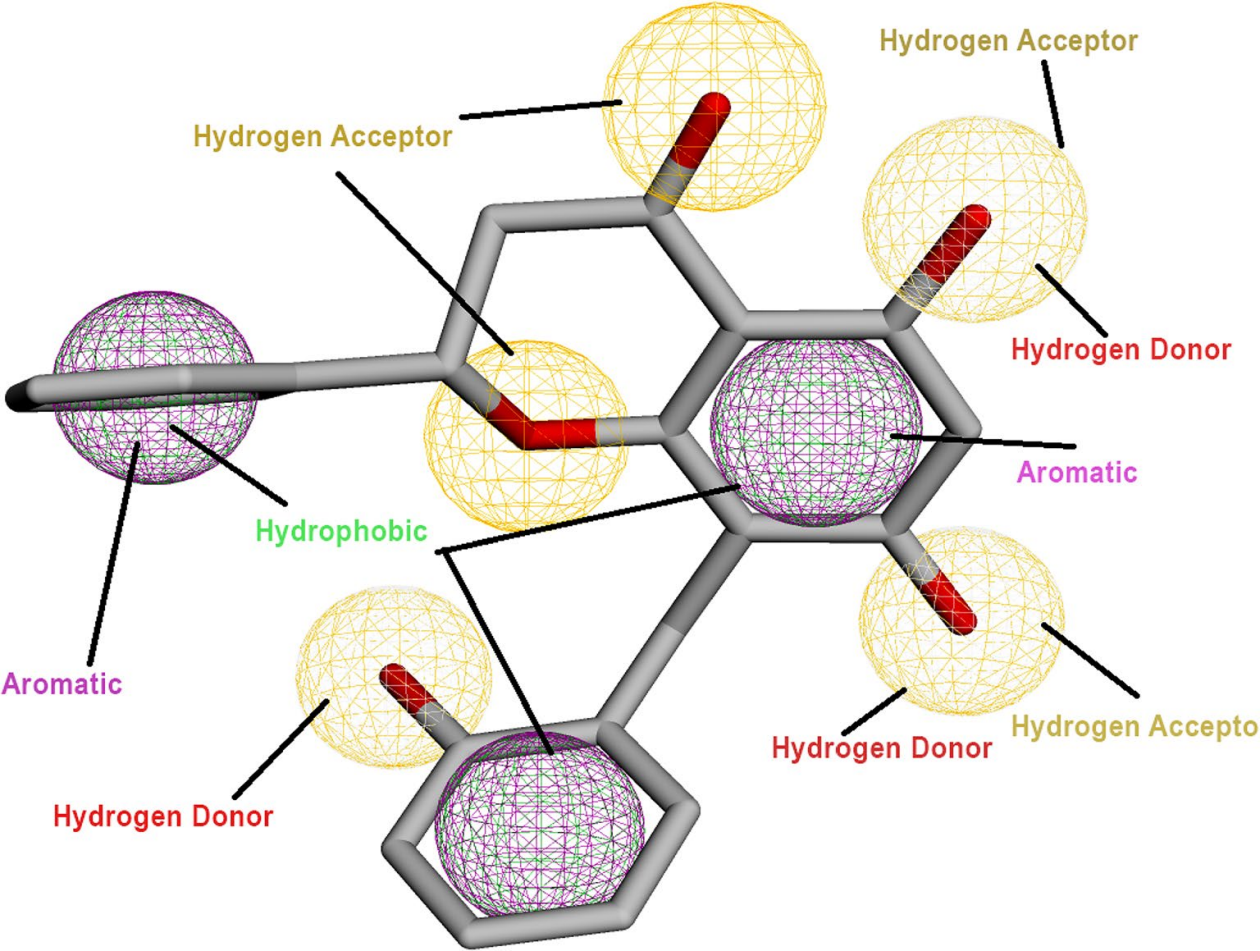
Pharmacophore characterization of chamanetin. The sites that can form bonds are seen in circles. These atoms (sites) form bonds with the receptor protein.

**Figure 5:**
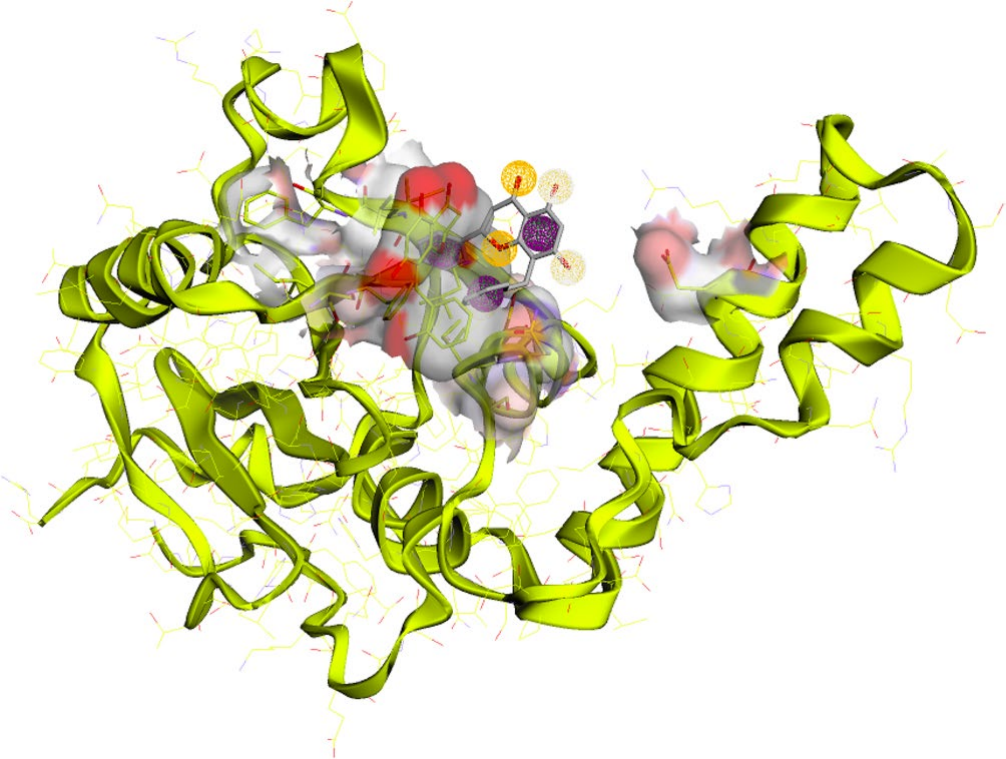
Pharmacophore characterization of dock-complex by pharmit.

The pharmacophore characterization of the ligand and dock complex can be seen in the Figures. chamanetin has 5 hydrogen acceptors, 3 aromatics, 3 hydrogen donors and 3 hydrophobic sites. The chamanetin was rich in pharmacophore sites and had good potential to bond with the receptor, forming a good number of interactions in Fig. 4. The RSMD table of pharmacophores from MolPort can be seen in Table 3. The pharmacophore of chamanetin docked with ATXN3 can be seen in Fig. 5.

**Table 3:** RSMD values of pharmacophore from MolPort database.

Name	RSMD	Mass	Rbands
Pharmacophore Results			
MolPort-042-675-535	0.275	468	5
MolPort-042-675-536	0.276	468	5
MolPort-042-675-537	0.301	468	5
MolPort-042-675-538	0.315	468	5

### ADMET analysis

ADMET analysis examined physiochemical parameters such as water solubility, GI absorption, skin permeability, and bioavailability score. Molinspiration Chemoinformatics predicts that the drug-likeness depicts no violations of Lipinski’s rule by chamanetin. The parametric values for chamanetin can be seen in Table 4.

**Table 4:** ADMET parameters for chamanetin.

ADMET parameters	Parametric values
Formula	C_16_H_26_O_5_
Molecular weight	308.45 g/mol
Num. heavy atoms	21
Fraction Csp3	1.0
Num. rotatable bonds	0
Num. H-bond acceptors	5
Num. H-bond donors	4
Molar Refractivity	76.34
TPSA (topological polar surface area)	90.15 Å^2^
Water solubility log *S *(ESOL)	− 1.60
GI absorption	High
Skin permeation (Log *K*_p_)	− 8.35 cm/s
Bioavailability score	0.55
Synthetic accessibility	5.37

### Boiled egg model

**Figure 6:**
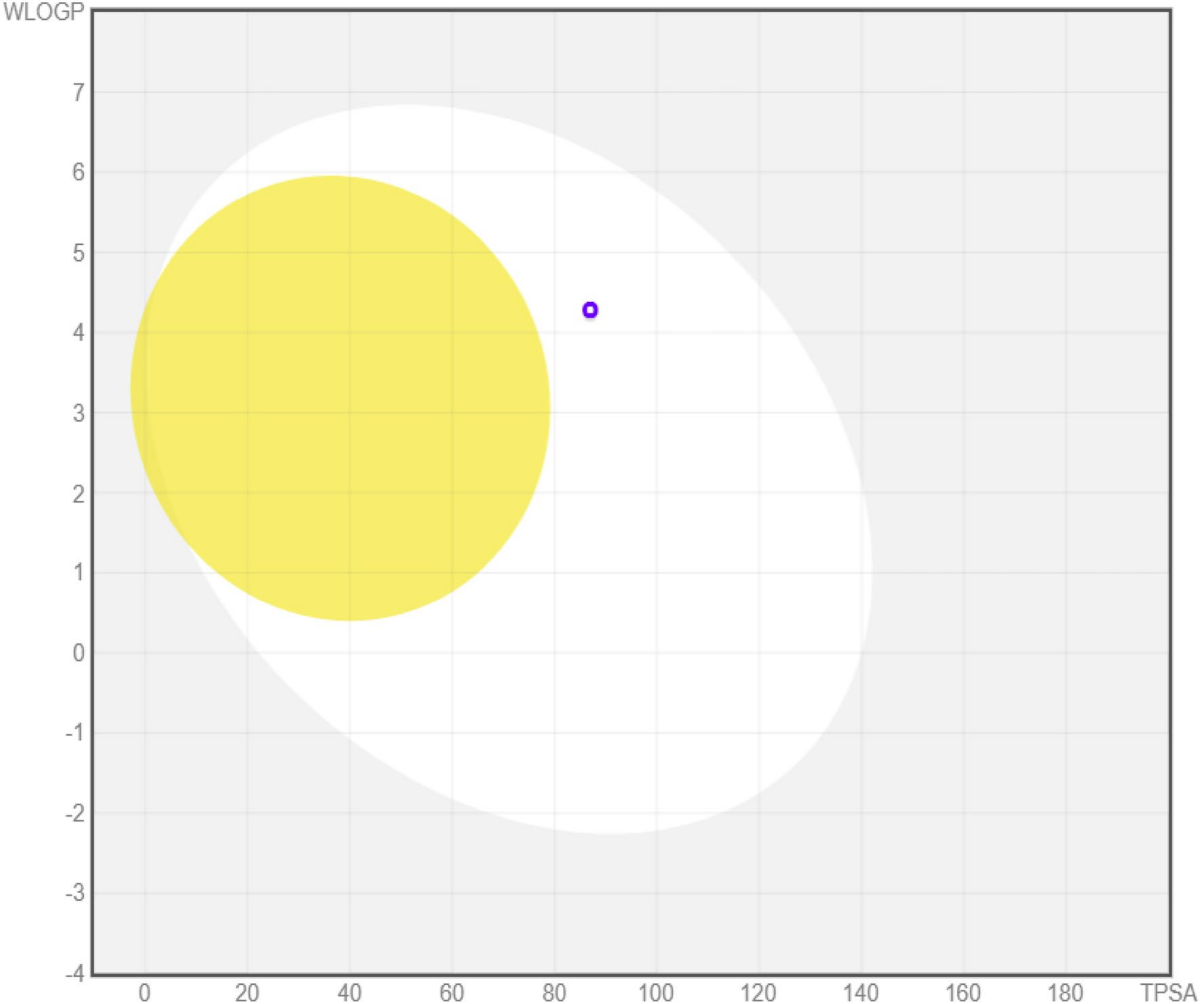
Boiled Egg diagram for Chamaetin. The yellow area depicts that molecules have access to the blood–brain barrier, while the white part human intestinal absorption for that predicted drug complex. Chamaetin has crossed the gastrointestinal track with a PGP + value.

The boiled egg model showed brain and gastrointestinal absorption, which was extremely helpful for creating and administering drugs. As shown in Fig. 6, the molecule is present in the white area, which predicts that chamanetin could cross the gastrointestinal absorption.

### Toxicity prediction

**Figure 7:**
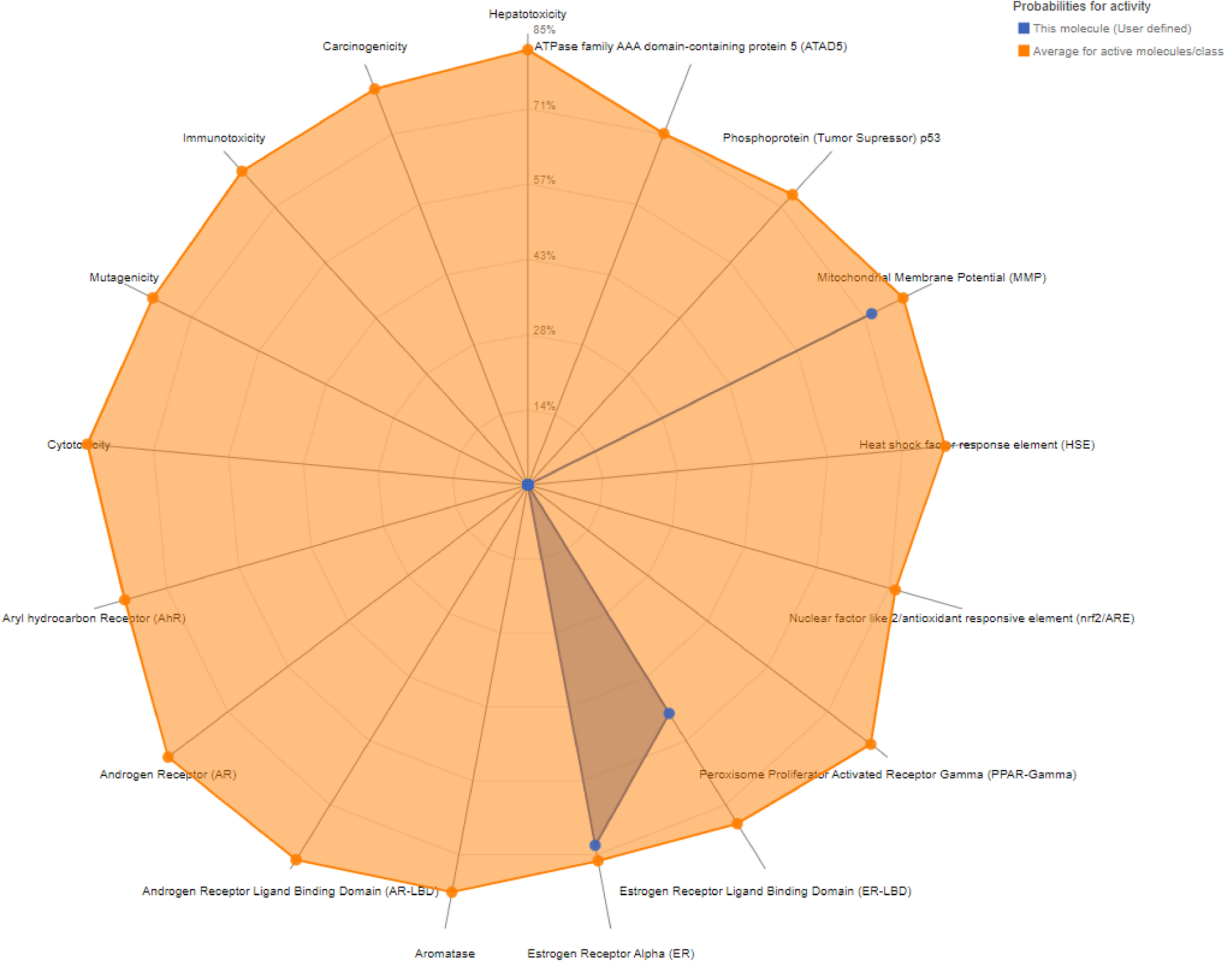
Prediction of toxicity of the Chamanetin by Pro Tox II. The red dots represent the inactive response of chamanetin, while the blue dots represent the inactive response of the chamanetin towards the receptor. The longer the yellow area is occupied in the chart graph, the more accurately the probability is predicted.

Chamanetin was nontoxic with no carcinogenicity, hepatotoxicity, immunotoxicity, cytotoxicity, mutagenicity, or other receptors. Estrogen Receptor Alpha (ER), Estrogen Receptor Ligand Binding Domain (ER-LBD), and Mitochondrial Membrane Potential (MMP) were the active toxicity receptors; however, these receptors play a less significant role in the development of toxicity in the body. The toxicity prediction can be seen in Table 5 and Fig. 7.

**Table 5:** Pro Tox II toxicity prediction of toxicity points and receptors for Chamanetin.

Classification	Target	Prediction	Probability
Organ toxicity	Hepatotoxicity	Inactive	0.72
Toxicity endpoints	Carcinogenicity	Inactive	0.71
Toxicity endpoints	Immunotoxicity	Inactive	0.72
Toxicity endpoints	Mutagenicity	Inactive	0.68
Toxicity endpoints	Cytotoxicity	Inactive	0.68
Tox21-nuclear receptor signaling pathways	Aryl hydrocarbon receptor (AhR)	Inactive	0.64
Tox21-nuclear receptor signaling pathways	Androgen receptor (AR)	Inactive	0.76
Tox21-nuclear receptor signaling pathways	Androgen receptor ligand binding domain (AR-LBD)	Inactive	0.99
Tox21-nuclear receptor signalling pathways	Aromatase	Inactive	0.75
Tox21-Nuclear receptor signalling pathways	Estrogen receptor alpha (ER)	Active	0.69
Tox21-nuclear receptor signalling pathways	Estrogen receptor ligand binding domain (ER-LBD)	Active	0.5
Tox21-nuclear receptor signalling pathways	Peroxisome proliferator activated receptor gamma (PPAR-Gamma)	Inactive	0.69
Tox21-stress response pathways	Nuclear factor (erythroid-derived 2)-like 2/antioxidant responsive element (nrf2/ARE)	Inactive	0.93
Tox21-stress response pathways	Heat shock factor response element (HSE)	Inactive	0.93
Tox21-stress response pathways	Mitochondrial membrane potential (MMP)	Active	0.72
Tox21-stress response pathways	Phosphoprotein (tumor supressor) p53	Inactive	0.54
Tox21-stress response pathways	ATPase family AAA domain-containing protein 5 (ATAD5)	Inactive	0.78

### Interaction analysis

**Figure 8:**
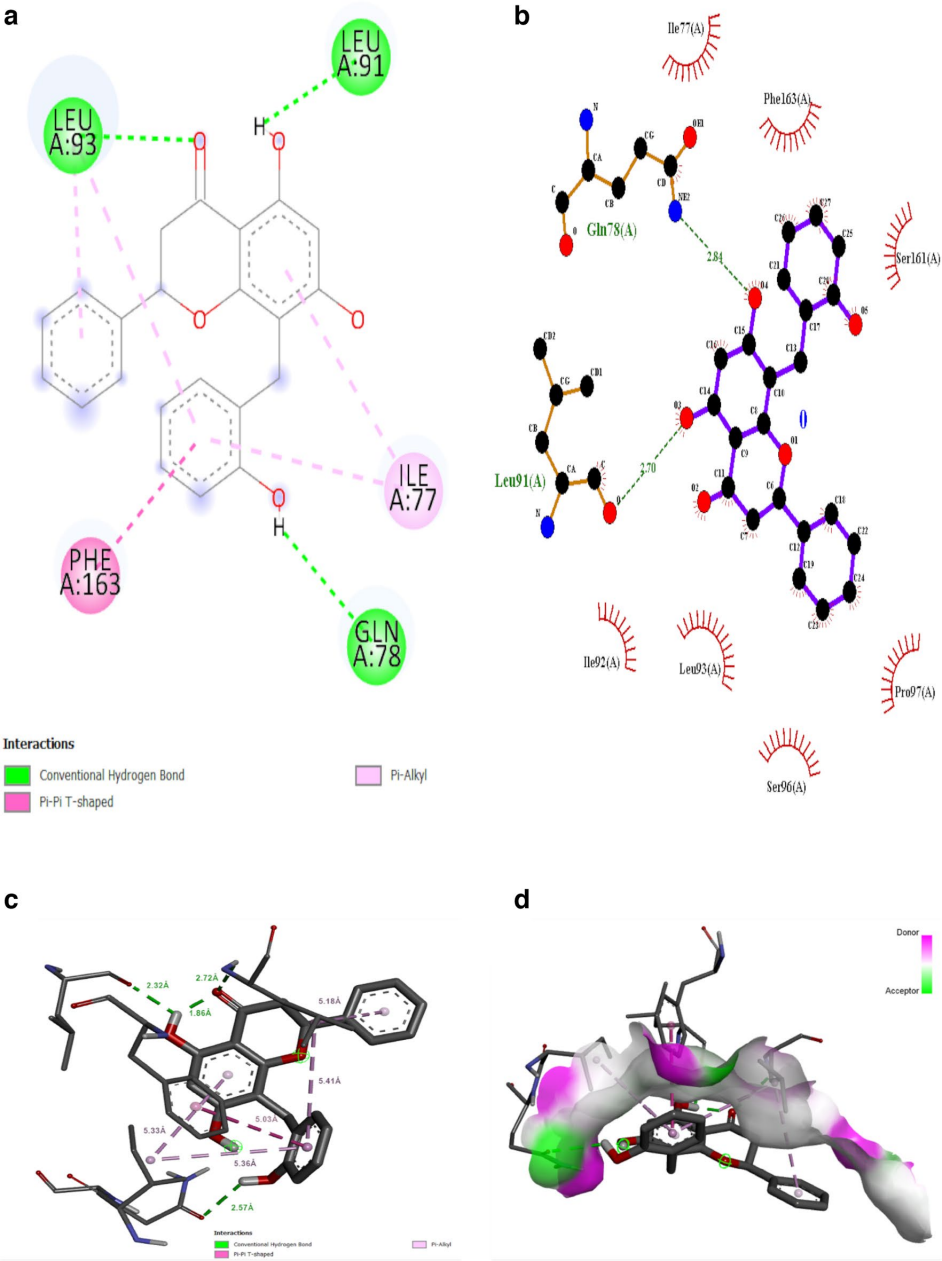

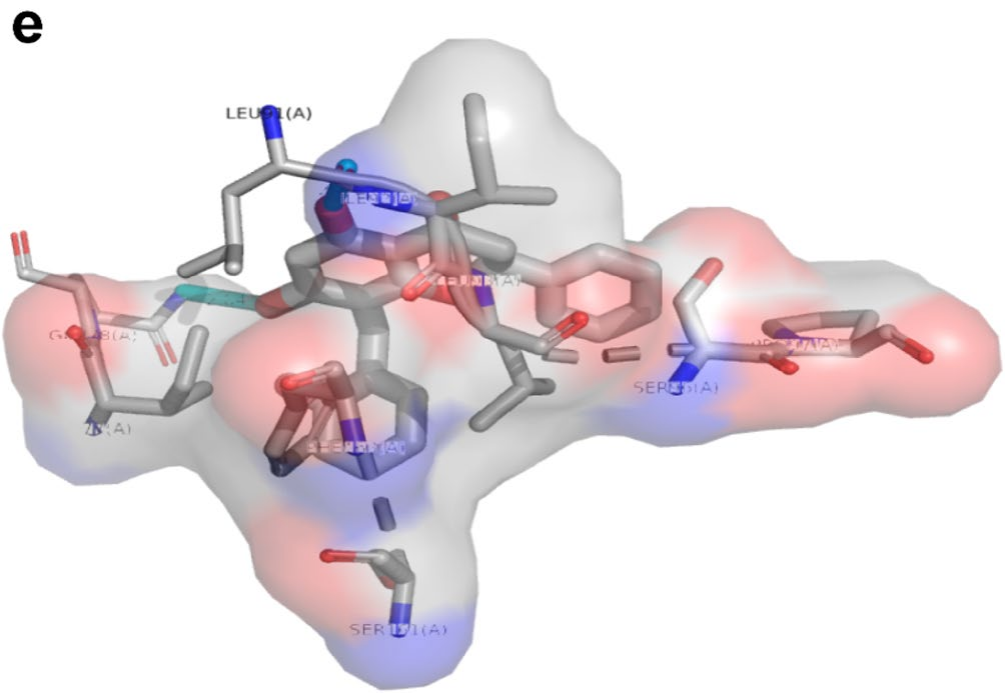
(**a**) 2D molecular interaction diagrams by Discovery Studio and Ligplot + . (**b**) The bond and bond lengths between chamanetin with ATXN3 can be seen in this diagram. (**c**) 3D molecular interaction between ATXN3 and Chamanetin by Discovery Studio. (**d**)The area of the dock complex where hydrogen ions can be accepted and donated. (**e**) Hydrophobic and hydrophilic interaction by pymol. The red-highlighted region is hydrophobic, and the blue-highlighted region is hydrogen-accepting.

Autodock vina was used to dock the phytochemicals with ATXN3 protein. Based on binding energy, the best compound with the lowest binding energy was Chamanetin. Chamanetin has a binding affinity of − 7.2 kcal/mol. Molecular interaction diagrams were generated through ligplot + . Chamanetin shows conventional hydrogen bonding with Leu93, Leu91 and Gln78 residues with a bond length of 2.32 Å, 1.86 Å, 2.57 Å and 2.72 Å. It also formed one Pi-Alkyl interaction with Ile77 having bond lengths of 5.33 Å, 5.36 Å and one Pi-Pi- T shaped interaction with Phe163 having bond lengths of 5.03 Å, 5.41 Å. Other residues that show indirect interactions were Ser161, Pro97, Ser96 and Il92. The 2D and 3D interaction diagrams can be seen in Fig. 8.

### Molecular dynamics simulations

**Figure 9:**
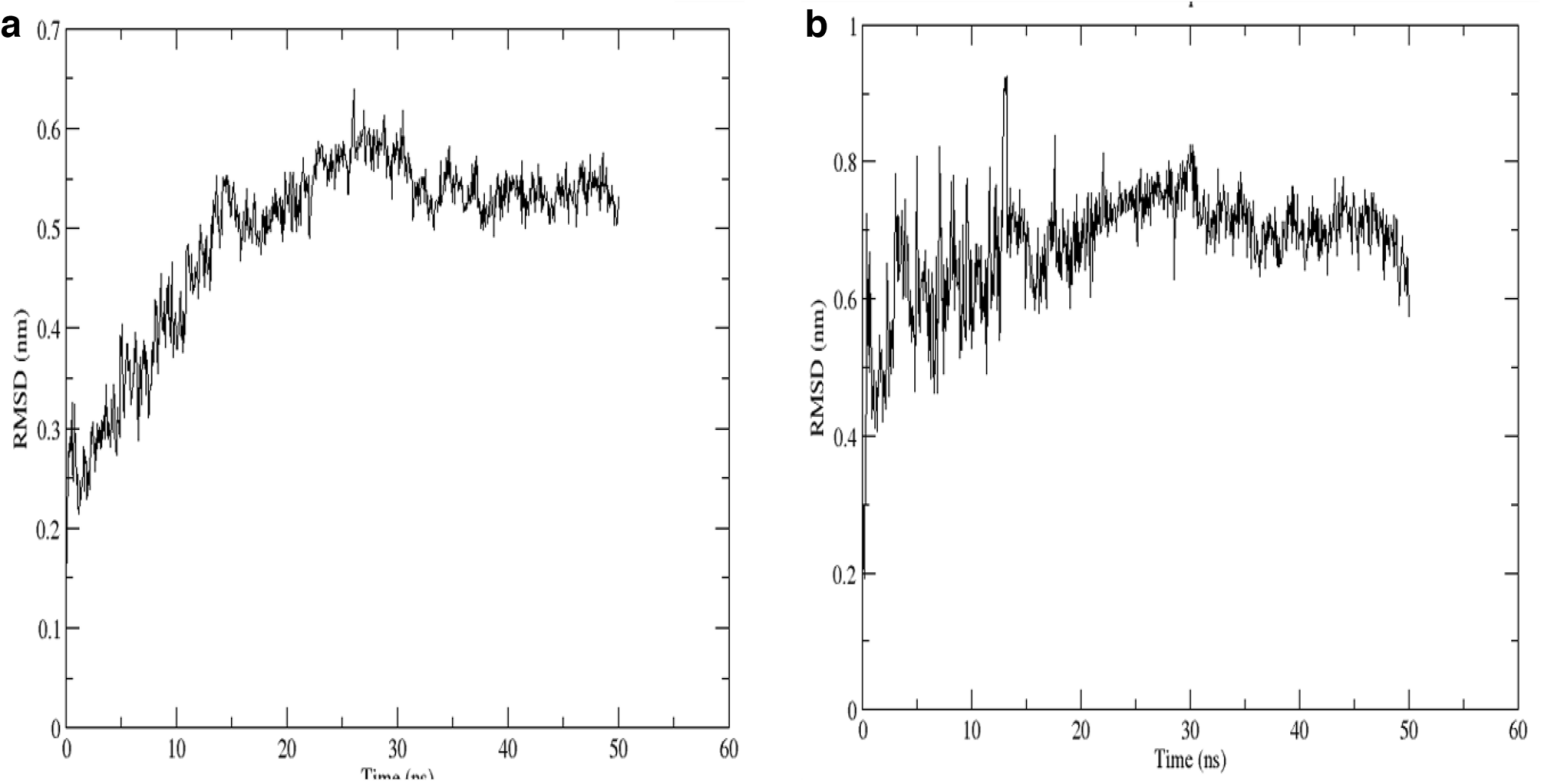
(**a**) RSMD of ATXN3 is increasing simultaneously till 30ns and stabilizes with the RSMD value of 0.6. (**b**) RSMD of Chamanetin ATXN3 increases simultaneously till 20ns and stabilizes with the RSMD value of 0.8. This shows good interaction between the ATXN3 and Chamanetin, and the simulation, in the end, shows a stable interaction.

**Figure 10:**
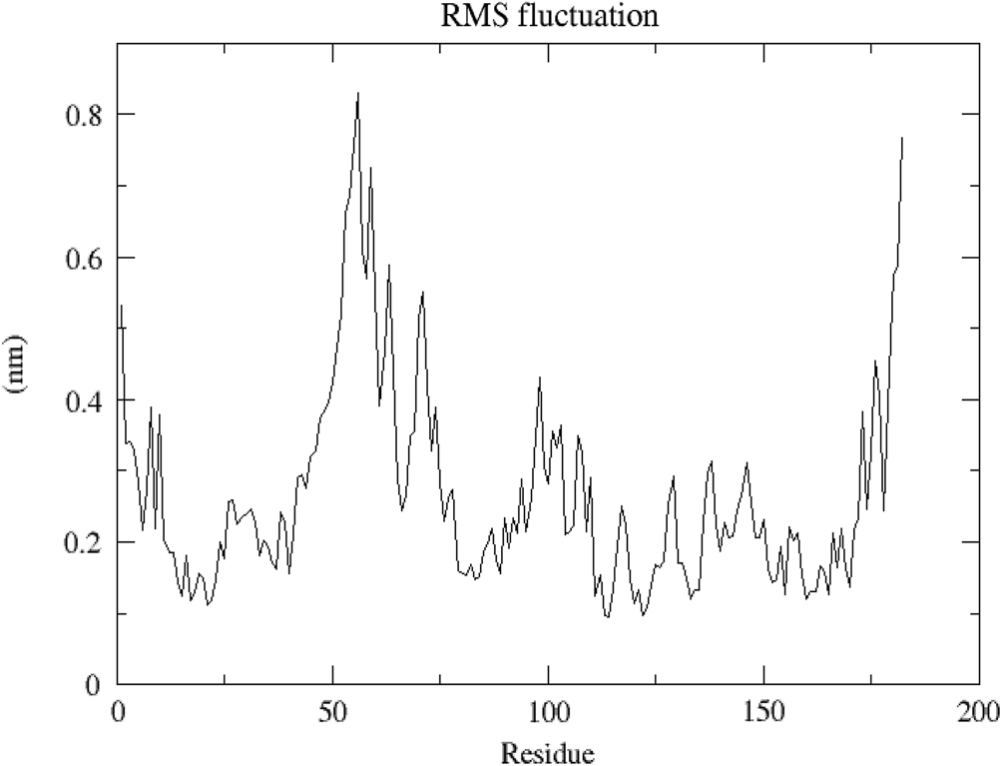
RMSF of ATXN3 by Gromacs shows fluctuation in the ATXN3. The amino acid shows much fluctuation from 50 residues to 75; after this, the residues show less fluctuation. After almost 160 amino acid residues, the protein amino acids show a big fluctuation.

MD 50 ns simulation was run on the dock complex of ATXN3 with Chamanetin. RSMD of the simulation showed that the protein and ligand were interacting and stabilized after 20 ns. The RSMD of the dock complex can be seen in Fig. 9. The RMSF of the protein showed that the fluctuation in the protein residues is normal and lies at 0.1–0.8, which is an average fluctuation score. The RSMF of the protein can be seen in Fig. 10.

## Discussion

Spinocerebellar ataxia type 3 stands as one of the most widespread hereditary neurodegenerative disorders. The causative gene, ATXN3, leads to the abnormal expansion of cytosine-adenine-guanine (CAG) trinucleotide repeats, producing a potentially harmful polyglutamine protein^27^. Gait ataxia emerges as the predominant symptom in SCA3, with an average lifespan of around 21.18 years following the onset of symptoms. Additional manifestations encompass hyporeflexia, peripheral neuropathy, atrophy of the distal muscles, and cerebellar ataxia. Regrettably, these symptoms progressively deteriorate over time, and there is no cure for the condition^28^. Despite extensive clinical trials exploring potential therapies, there is no FDA-approved medication for SCA3. Contemporary therapeutic approaches involve supportive care and physical therapy to address motor dysfunction, targeting specific active toxicity receptors such as estrogen receptor alpha (ER alpha), estrogen receptor ligand binding domain (ER-LBD), and mitochondrial membrane potential (MMP) symptoms. To advance treatment strategies, more comprehensive screening of biological markers in presymptomatic and symptomatic patients is essential to identify novel target molecules and understand pathogenic pathways. Unfortunately, no existing or investigational medications appear poised to bring about imminent changes in SCA3 treatment^29^.

5-HT1A receptor agonists were the initial drugs investigated in clinical trials for SCA3 therapy, and early studies of varying sizes yielded promising results^9^. In a 2012 study conducted in Taiwan, allogeneic adult adipose-derived mesenchymal stem cells (MSCs) were utilized to treat SCA3. These cells were chosen for their demonstrated ability to safeguard neurons through trophic factor synthesis and the reduction of reactive oxygen species (ROS) generation. Importantly, allogeneic cells were preferred over autologous cells due to their resilience against potential ineffectiveness arising from underlying genetic issues^30^. In a separate study, the efficacy of growth hormone therapy was explored. However, it is noteworthy that this therapeutic approach carries a minor risk of cancer when administered over an extended period^31^. Flavonoids, a category of phytochemicals present in numerous plant-based foods like fruits, vegetables, grains, and herbs, are renowned for their potential health benefits. Their recognized antioxidant, anti-inflammatory, and anticancer properties have spurred significant research interest. The diverse bioactive compounds present in Melia azedarach, are employed molecular docking and QSAR analyses to predict favorable interactions with Mycobacterium tuberculosis proteins^13^. Preliminary results demonstrate promising binding affinities, highlighting the feasibility of utilizing natural compounds for expedited drug discovery against tuberculosis^13^. Typically considered safe when consumed in dietary amounts, flavonoids have been explored as a potential avenue for drug design against SCA3 using an in-silico research approach^32^.

In the docking analysis, the screening of flavonoids focused on potential interactions and attachments to the 1YZC target. While existing drugs have demonstrated limited effectiveness, specifically targeting ATXN3, this study identified a promising candidate for spinocerebellar ataxia type 3. A halogen engineering-based drug against Friedreich’s ataxia, employing a strategy akin to the one used in a previous study. By leveraging halogen engineering principles, they aim to enhance the therapeutic efficacy of the drug, offering a comprehensive approach to address the challenges associated with Friedreich's ataxia^33^. Chamanetin, among the screened flavonoids, exhibited robust binding energies, specifically − 7.2 kJ/mol, suggesting its potential to mitigate and possibly eradicate SCA3 with good ADMET, Pharmacophore and interaction studies. To overcome multiple psychological issues and deaths a candidate drug with more potential has been designed.

## Conclusion

According to the results of this study, ATXN3 is involved in the progression of many neurological diseases and mental health problems in a special way. The prevalence of spinocerebellar ataxia type 3 is on the rise, and there is currently no FDA-approved treatment for this condition. This type 3 spinocerebellar ataxia patient needs medicine for an hour so they can get better. According to concerns, the overexpression of ATXN3 may promote the abnormally enlarged polyglutamine. As a result, we have developed a medication to treat type 3 spinocerebellar ataxia using phytochemicals that target the ATXN3 protein. One potential therapeutic candidate against ATXN3 was the flavonoid chamanetin. When docked to the active site of ATXN3, Chamanetin exhibited a strong affinity according to the MD docking and simulation data. The findings indicate that the pharmaceutical candidate is non-toxic and pharmacophore and does not break Lipinski’s criteria.

### Future prospective

The proposed study is limited to the in-silico analysis, which supports the idea that the phytochemicals from plant sources would be a good therapeutic target for SC3, but these findings must be validated in vitro and in vivo analysis.

## Data Availability

All the data generated in this research study has been included in this manuscript.
